# Adaptive Quasi-Unsupervised Detection of Smoke Plume by LiDAR

**DOI:** 10.3390/s20226602

**Published:** 2020-11-18

**Authors:** Riccardo Rossi, Michela Gelfusa, Andrea Malizia, Pasqualino Gaudio

**Affiliations:** 1 Department of Industrial Engineering, University of Rome “Tor Vergata”, Via del Politecnico 1, 00133 Rome, Italy; R.Rossi@ing.uniroma2.it (R.R.); gelfusa@ing.uniroma2.it (M.G.); 2 Department of Biomedicine and Prevention, University of Rome “Tor Vergata”, Via di Montpellier 1, 00133 Rome, Italy; malizia@ing.uniroma2.it

**Keywords:** LiDAR, fire detection, machine learning, automatic detection, SVM

## Abstract

The early detection of fire is one of the possible applications of LiDAR techniques. The smoke generated by a fire is mainly compounded of CO_2_, H_2_O, particulate, and other combustion products, which involve the local variation of the scattering of the electromagnetic wave at specific wavelengths. The increases of the backscattering coefficient are transduced in peaks on the signal of the backscattering power recorded by the LiDAR system, located exactly where the smoke plume is, allowing not only the detection of a fire but also its localization. The signal processing of the LiDAR signals is critical in the determination of the performances of the fire detection. It is important that the sensitivity of the apparatus is high enough but also that the number of false alarms is small, in order to avoid the trigger of useless and expensive countermeasures. In this work, a new analysis method, based on an adaptive quasi-unsupervised approach was used to ensure that the algorithm is continuously updated to the boundary conditions of the system, such as the weather and experimental apparatus issues. The method has been tested on an experimental campaign of 227 pulses and the performances have been analyzed in terms of sensitivity and specificity.

## 1. Introduction

LiDAR techniques are laser-based remote sensing methods widely used for the study of the atmosphere. The techniques are based on probing the atmosphere by a laser beam and on analyzing the backscattering signals at specific wavelengths [1,2]. They are meant to study the vertical property profile of the atmosphere, providing density and temperature information, chemical gas and particulate compositions, and more [3,4,5]. In recent years, LiDAR-based methods are finding many other environmental applications, such as pollution monitoring in urban and industrial areas and early detection of fire [6,7]. 

The smoke plume developed by a fire is mainly compounded by CO_2_, H_2_O, particulate, and other minor products of the combustion. When the laser beam crosses the smoke plume, both the scattering and the absorption of the electromagnetic wave increases [8,9]. This effect is directly observed on the intensity of the backscattered radiation measured by a LiDAR apparatus, which reports a peak where the smoke is located [10]. Previous studies demonstrated the capability of LiDAR to be an effective technique for early detection and localization of smoke and fire, helping in avoiding the increase of fire sizes and severity, and decreasing the critical consequences that may affect the environment [11,12]. The efficiency and limits of this technique to smoke fire detection are strongly related to different parameters of the system itself (laser pulse properties, receiver, etc.), the distance, characteristics of the smoke plume, and environmental conditions (wind, fog, etc.). A sensitivity analysis of this technique was analytically/numerically investigated by Bellecci et al. [13].

Despite it being demonstrated that LiDAR technique is a sensitive method for the early detection of fire, the analysis of the signals needs caution, especially to avoid a high number of false alarms, because LiDAR signals sensitive to several atmospheric variables. The most common approach to detect fire is to use peak-detection algorithms, since the presence of smoke leads to an increase of the local extinction coefficient. However, they are not so performant. A peak amplitude threshold must be used to ensure a small number of false alarms, losing sensitivity. A few alternative approaches, such as machine learning-based techniques, were developed and tested, and they seemed to provide better results [14]. The use of the LiDAR in real-time applications poses some issues on the best selection of the parameters and the most critical aspect is the unsteadiness of the application. In fact, the continuous monitoring of an area, day by day, season by season, involves that the environment changes. The weather, the air temperature, density, and water vapor concentration vary, thus, involving a shift of the best parameters of the algorithms. This work aims to present and test a new LiDAR analysis approach for the early detection of fires. The new technique is based on an unsupervised classification method designed for this application, which has the advantage to be self-calibrating. Moreover, in order to face the unsteadiness of the application, an adaptive calibration logic has been adopted, ensuring the best performances at any time.

## 2. Materials and Methods

### 2.1. LiDAR Apparatus and Measurement Scheme

**Figure 1:**
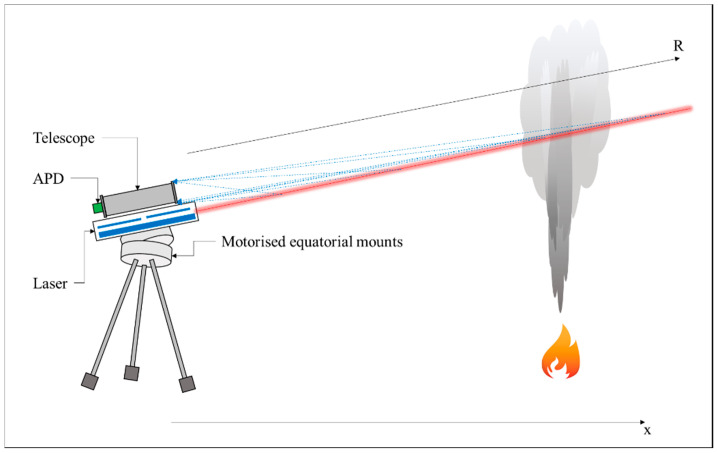
Scheme of the experimental apparatus.

Experimental measurements were performed to analyze and validate the new method. The experimental apparatus is a compact and robust mobile LiDAR system based on a monostatic biaxial configuration, where the laser and the telescope were placed together on the same structure, but have different axes. The laser was a pulsed Q-switch Nd:YaG (Quantel Laser (now LUMIBIRD), Lannion, France) operating at the fundamental wavelength of 1064 nm. The maximum repetition rate was 10 Hz and the pulse was 8 ns. The beam diameter was about 5 mm, and the beam divergence was lower than 4 mrad. “In order to avoid dangerous radiation in the environment, it has been chosen to attenuate the laser radiation, by using a laser beam attenuator with an attenuation factor of 10, involving that the laser beam fluence is under the safety fluence limit of 20 mJ/m^2^ at one distance of 200 m”. The backscattered light was collected by the telescope (it is a commercial Cassegrain), with a nominal focal length of 1300 mm, a primary mirror diameter of 102 mm, an f-number of f/12.7, and a field of view of 1.2 mrad. The signal was detected by an IR-enhanced Avalanche PhotoDiode (APD, Hamamatsu Photonics K.K., Ammersee, Germany), which had a response time lower than 5 ns, a gain of 100, and a quantum efficiency of 38%. A narrow bandpass filter centered at 1064 nm and wide 2 nm was used to filter background radiation. Then, the signal was recorded by a National Instrument PXI (NATIONAL INSTRUMENTS CORP., Austin, TX, USA), which used a card NI-PXI 5122 with a resolution of 14 bit and a sampling rate of 100 Msamples/s. The measurements were performed producing a controlled fire, at a distance of about 400 m from the LiDAR equipment. Measurements were taken without a specific measurement frequency, but accordingly to the change of the fire. At first, pulses with no fire were recorded. Then, the fire was made and other measurements were performed. This process was reapplied three times, leading to a number of measurement equal to 227, 61 safe, and 166 with fire at different intensities. Table 1 resumes the characteristics of the main components of the LiDAR system, while Figure 1 shows a schematic view of the system.

### 2.2. LiDAR Inversion

The LiDAR technique probes the atmosphere by a laser beam in the infrared, visible, or ultraviolet region and analyses the backscattered power signal. A LiDAR can use different types of radiation, which is a function of the laser wavelength and the filtering of the signal. When the radiation wavelength is the same as the laser wavelength, the LiDAR is a Rayleigh-Mie based LiDAR which used the “elastic scattering”. The backscattered power can be written as follows [15]:

(1) $$P\left(r\right)=\frac{C_{0}\beta_{\pi }\left(r\right)}{r^{2}}\exp\left(-2\int_{0}^{r}\alpha \left(z\right)\mathrm{dz}\right)+B$$

where P(r) is the backscattered power, r is the distance, C_0_ is a constant of the system, β_π_ is the backscattering coefficient, k_t_ is the extinction coefficient, and B is the background radiation. The background radiation can be easily removed from the signal just measuring the background radiation (laser off).

The LiDAR equation contains two unknowns, the backscattering and the extinction coefficient. Different methods, based on approximations or atmospheric assumptions, were developed to calculate the backscattering coefficient from the LiDAR equation, such as the slope method, the boundary point solution, and the optical depth solution. In this work, the similar approach presented by Klett [16,17] was used. The method is based on assuming that there is a correlation between the volume extinction and the backscatter. If this correlation is known, Equation (1) has only one unknown and the backscattering coefficient can be calculated:

(2) $$\beta_{\pi }\left(r\right)=\frac{\exp\left(S\left(\lambda ,r\right)-S_{m}\left(\lambda \right)\right)}{\frac{1}{\beta_{\pi ,m}}+\frac{2}{B_{p}}\int_{r}^{r_{m}}\exp\left(S\left(\lambda ,\;R^{\prime }\right)-S_{m}\left(\lambda \right)\right)d\;r^{\prime }}$$

where S(λ,R) is $P\left(r\right)r^{2}$, $S_{m}\left(\lambda ,R\right)$ is calculated at the “reference” position, the $\beta_{\pi ,m}$ is the backscattering radiation at the reference position. B_p_ is the “phase function”), calculated as the ratio between β and α. This approach requires that you know the phase function and the backscattering at the reference position. In our case, the phase function was considered constant along the path and equal to 0.43. The reference backscattering coefficient was taken as equal to 1.047 × 10^−4^ at the reference position of 200 m (the values have been calculated from Shettle et al. [18]). It has to be mentioned that the method used in this work was quite simple and based on assumptions that may not be always satisfied and that in the literature there can be found more robust approaches, even a data-driven one, which minimizes the intervention of the operators. However, the eventual miscalculation of the absolute value of backscattering radiation is not so important in this work, being the aim of this work, the automatic detection of off-normal backscattering radiation respect with a standard measurement (see Section 2.3.).

Moreover, the moving standard deviation of the backscattering coefficient was applied, and it was used as input of the classification model to increase the performances of the algorithm. The standard deviation was computed by a central moving standard deviation in a sliding window of 30 m.

The signals used for the inversion and standard deviations are the results of only one laser pulse, thus, no signal averaging has been used and it means that each LiDAR profile corresponded to one laser pulse.

### 2.3. Quasi-Unsupervised Classification Algorithm

The core of the classification algorithm is the support-vector machine (SVM). The SVM is a supervised algorithm that, by giving a certain number of examples, where both inputs and outputs are known, creates a model where the operative spaces of each class are determined. An SVM is aimed at generating one or more hyperplanes that allow to separate the points of the classes. The simplest SVM is the linear SVM, which generates a plane with the input variables that maximize the classification output. However, the linear SVM is usually not sufficient and high-order hyperplanes are generated using a different kernel function (most common are parabolic, cubic, gaussian). Once the hyperplane is found, the model is used to determine the class of new points, where their classes are unknown [19]. In the present work, the class should be “alarm” and “safe”. SVM, such as any other supervised classifier, was strongly affected by the training set. The LiDAR returns the backscattered coefficient as a function of the time (or distance) and, since the smoke is local, a no safe profile contains both “safe” and “alarm” points. Moreover, due to wind, the “manual” localization of the smoke may be affected by uncertainties, and a supervised training may be hazardous, involving that also the training set may be affected by errors. Moreover, an adaptive supervised algorithm would require the continuous involvement of an operator that must classify the pulses for the training set update, making that application not automatic. Thus, an alternative solution was found using a one-class SVM [20], where the class is the “safe” one. The safe and alarm classification by the one-class SVM was obtained by a probability threshold. In brief, the SVM returns the probability that the analyzed point belongs to the same population of the training set class (safe). Then, the probability was converted in a Z_score_ by the inversion of gauss function. The kernel function used was a gaussian kernel.

**Figure 2:**
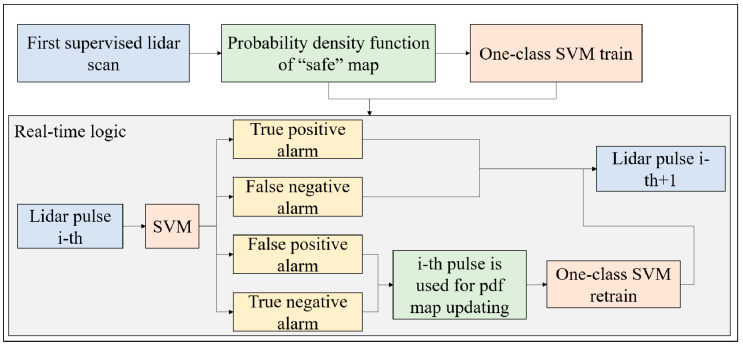
Schematics of the classification algorithm logic. Abbreviations: SVM, support-vector machine.

The logic of the algorithm is shown in Figure 2.

At first, a first LiDAR scan was performed in the area, when there is no smoke and fire. Then, the probability density function maps of both backscattering and backscattering standard deviation as a function of the distances were calculated. For any bin of the distance map, N number of points were generated following the pdf at that distance and the SVM was trained. So, the SVM was ready for the real-time application. The i-th laser pulse was classified by the SVM and there were four different possibilities:

1. True positive alarm: the SVM classifies an alarm, the operator takes the countermeasures and confirms the presence of smoke and fire. In this case, the LiDAR continues the monitoring and goes to the i-th + 1 pulse.
2. False positive alarm: the SVM classifies an alarm, the operator takes the countermeasures and negates the presence of smoke and fire. In this case, the data of the i-th pulse, which is a “safe” pulse, is used to update the map and retrain the SVM.
3. True negative alarm: the SVM does not classify an alarm, no fires and smokes are observed in the following minutes. The pulse is a “safe” pulse and it is used to update the map and retrain the SVM.
4. False negative alarm: the SVM does not classify an alarm, but within the next minutes, smokes and fires are observed. The pulse is not a “safe” pulse and cannot be used to update the map and retrain the SVM.

## 3. Results

**Figure 3:**
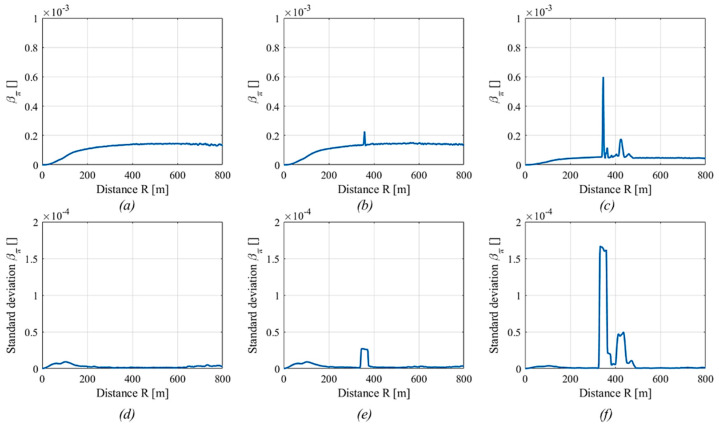
Backscattering coefficient amplitude (**a**–**c**) and standard deviation (**d**–**f**) as a function of the distance in the case of no smoke (pulse 10, **a** and **d**), small smoke plume (pulse 15, **b** and **e**), and large smoke plume (pulse 21, **c** and **f**).

Figure 3 shows the backscattering coefficient amplitude and moving standard deviation in three different cases.

On the left, a pulse with no smoke is present. The backscattering coefficient amplitude was almost flat, excluded in the first 200 m, due to the configuration of the experimental apparatus (it was a biaxial LiDAR and the telescope is normally blind in the first meters). The backscattering standard deviation was almost flat too, there was a small rise in the first 200 m due to the increase of the backscattering amplitude, a slight increase at the end of the pulse, since the SNR of the signal decrease and the uncertainty arise. A pulse with a small amount of smoke along the laser path was shown in the center. Here, a smoke plume was placed at 375 m. Both the amplitude and standard deviation signals show an increase of their values where the smoke was placed and the moving standard deviation returned a wider window where the signal increased, which may help in increasing the detection of the fire in noisy signals. The last column of images shows a pulse where the fire was increased and, thus, the smoke plume also expanded. Thus, both the signals increased in the fire position.

**Figure 4:**
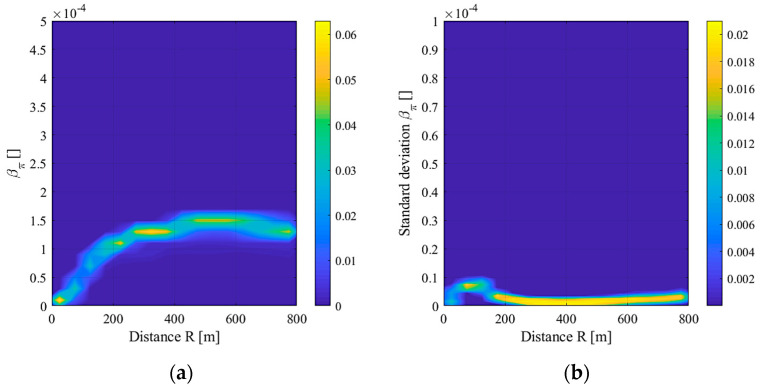
Probability density map of backscattered coefficient amplitude (**a**) and standard deviation (**b**).

As explained in the method section, the new algorithm was based on generating a probability density map (which are probability density functions at any distance) of the “safe” pulse, where no smoke is present. The maps for both the amplitude and standard deviation of the backscattering coefficient are shown in Figure 4.

The “safe” region, represented by the yellow-green-light blue colors, symbolizes the operative space where it is expected to have the points of the next LiDAR profiles (backscattering and moving standard deviation) if the pulse is a safe one. Contrariwise, if a smoke plume is present, the point should go out from the “safe” region. The narrow profiles indicate that both the laser functioning and weather conditions are stable during the measurements. In fact, if the laser pulse was unstable or a strong change of the atmosphere was present, the safe region map would have been wider, since each safe pulse would have shown different values of the backscattering coefficient and its standard deviation. During long experimental campaigns or real-time applications, this may not be true, especially due to natural changes, such as the weather. The introduction of adaptive logic avoided this problem, creating a wide map of safe pulses, ensuring a small number of false alarms.

**Figure 5:**
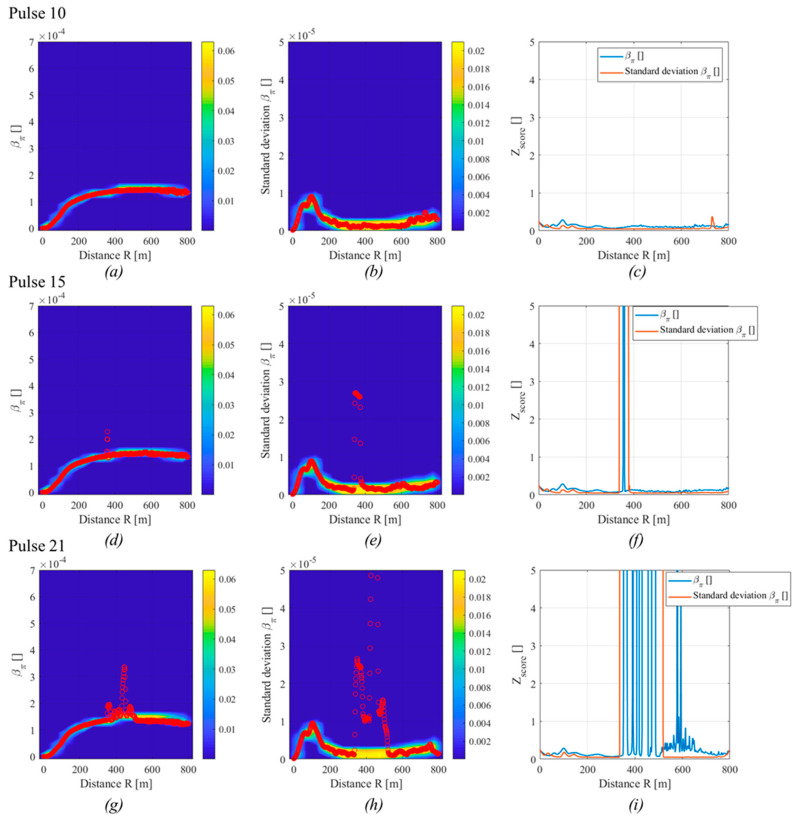
Signals of the backscattered coefficient amplitude (**a**,**d**,**g**) and standard deviation (**b**,**e**,**h**) on the pdf map with the signal value in red circles and signals of the Z_score_ (**c**,**f**,**i**) for the pulses 10 (**a**–**c**), 15 (**d**–**f**) and 21 (**g**–**i**).

The application of the maps on fire and smoke detection is shown in Figure 5.

Any image row of the figure shows one of the previous pulses (10, 15, and 21). The points of the safe pulse (pulse 10) lay on the safe region of both maps and the Z_score_ of the was lower than 1 at any distance, which suggested that there are not “alarm” points. The pulse 15, which had a small smoke plume at 375 m, shows that all the points lie on the safe region and excluded the points near 375 m. The Z_score_ of both signals increased in that region, with a wider area in the case of the standard deviation. The last pulse, where the smoke was well developed, is shown in the last row. Again, both the signals got out from the safe region where the fire was located. However, two different issues arise for the backscattering coefficient amplitude. The first is that the Z_score_ has several oscillations in the smoke region, while the standard deviation is better defined. Moreover, the backscattering amplitude has high Z_score_ also for larger distances, involving a difficulty in smoke detection. The last issue arose because of the interference of smoke with the LiDAR equation inversion, which slightly underestimated the backscattering amplitude, which goes in the “unsafe” region.

**Figure 6:**
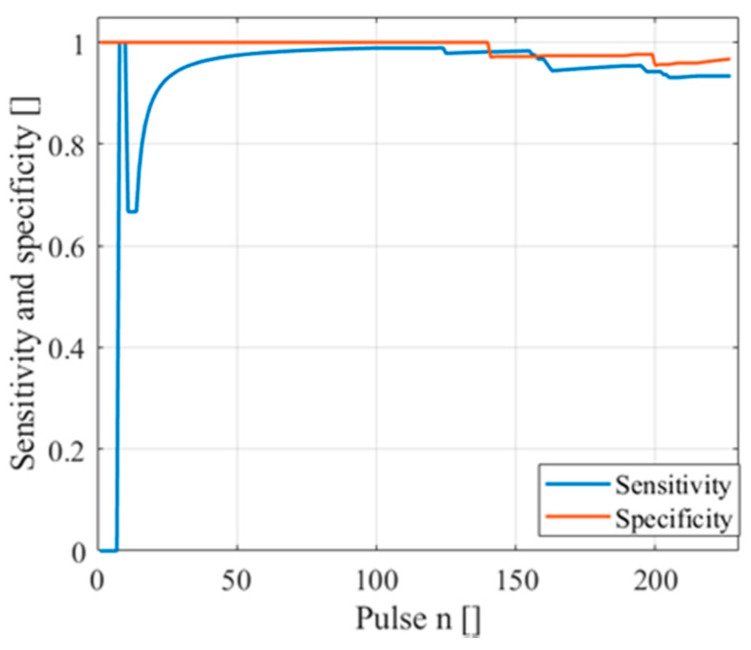
Sensitivity and specificity of the predictor in an experimental campaign.

The algorithm was tested on an experimental campaign of 227 pulses, 61 safe and 166 unsafe, with different levels of smoke, from low to high. The best results in terms of true positive rate and true negative rate, or sensitivity and specificity, have been obtained using the standard deviation signals, which, as discussed before, demonstrated to be more stable and less influenced by errors on the backscattering coefficient calculation. Figure 6 shows the trend of the specificity and sensitivity during the experimental campaign, using the real-time application. The trend was stable for both parameters. At the end of the campaign, the specificity was 96.7% (2 false alarms) and the sensitivity was 93.4% (11 missed alarms).

**Figure 7:**
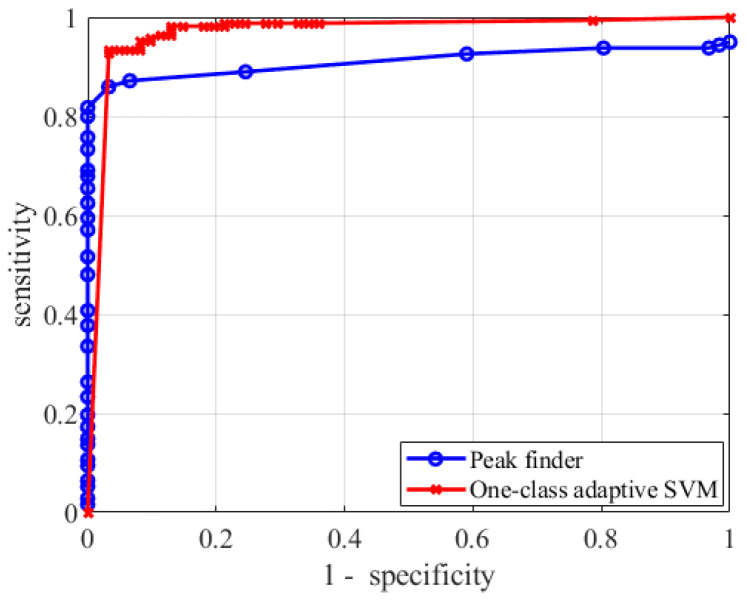
Receiver Operating Characteristic (ROC) curves of the peak finder algorithms and the new One-class adaptive support-vector machine (SVM).

The results obtained with the new algorithm were compared with the old peak finder algorithm. The peak finder was based on calculating the presence and the position of one or more peaks in a signal and the function used in this work is the one implemented in MATLAB, which allows to set the threshold, prominence, peak width, etc. When our experimental apparatus was built years ago, the peak finder algorithm was tested, and the best parameters were obtained leaving the default parameters of the peak finders and varying the peak prominence. The same algorithm was tested on the data used in this work and a parametric test was conducted varying the prominence value. The blue line in Figure 7 shows the Receiver Operating Characteristic (ROC) curve (specificity vs. sensitivity) of the algorithm (where each point is obtained using one prominence value). What can be observed is that:

1. The sensitivity does not reach very high performances, even for very small specificity (and thus a high number of false alarms);
2. The specificity is very high only for very small sensitivity;
3. The algorithm is very unstable in the “best performances” region, i.e., where both specificity and sensitivity are large enough (>80%). A variation of the 5% of the prominence lead to a variation of the performances of 10%, making the choice of the prominence very critical.

A similar analysis was conducted on the one-class adaptive SVM algorithm varying the Z_score_ value (from 10^−3^ to 10^2^, with a logarithm spacing) and the results are shown in the red line of Figure 7. The red line clearly shows that the SVM algorithm ensured higher sensitivity with same false alarms. For example, if we allow a maximum number of false alarms equal to 5%, we will have a sensitivity for the peak finder algorithm of about 86%, while the SVM algorithm returns a 93.4% (+ 7%). At last, it has been found that the algorithm was very robust on the optimal performance region.

## 4. Discussion and Conclusions

In this work, a new method for the analysis of LiDAR signals for the early detection of fire was introduced. In the presence of smoke, the local properties of the atmosphere changed and by probing it through a laser beam, it was possible to detect these differences and provide an alarm, in order to take the proper countermeasures and avoid fire growth and threats to the environment.

The new method consisted of a quasi-unsupervised approach, with an adaptive and real-time logic. The probability density function map of the backscattering coefficient amplitude and standard deviation were both used. The map, representing the “safe” region, allows to extract the points that better represent the population of the safe points, where there is no smoke. Then, a supervised algorithm, in this case a support vector machine, was used to generate a model that returns the probability of a point to belong to the safe population. Then, this probability was converted in a Z_score_ and by a threshold, each point was classified as “safe” and “alarm”.

The adaptive logic ensures that changes to the environment and experimental apparatus properties, which may change the backscattering coefficient amplitude and standard deviation, were continuously updated with new “safe” pulses.

The use of the backscattering standard deviation led to higher results in terms of both sensitivity and specificity. It was expected given the trends of the standard deviation on safe and alarm pulses, which were less noisy and affected by less issues related to the LiDAR equation inversion.

The number of missed alarms were 11 out of 166 (specificity equal to 93.4%), while the false alarms were 2 of 61 (specificity equal to 96.7%). Each output was the result deriving from one laser pulse and one backscattering profile. However, due to the high repetition rate of lasers, signal averaging or consistency alarm detection may be used to improve the performances. For example, using a laser with a repetition rate equal to 1 Hz, the user may perform 60 pulses in a minute and giving an alarm based on a statistic of the 60 pulses, helping in decreasing the false alarms in significant ways. Otherwise, an aerial scan may be performed, and the detection may be performed on the area (also creating a three-dimensional probability density function map). Using a 2D scansion, for example using the equatorial mounts, may also help in developing more sophisticated and accurate detection algorithms, even if they are probably slower. An example could be the approach used with the CALIPSO LiDAR [21]. However, any possible improvement of this approach is dependent on the applications and it could also be easily applied to other LiDAR applications, such as polluted area detection, etc.

The new algorithm was also compared with the older algorithm, based on a simple peak finder. It was found that the new adaptive one-class SVM ensures much higher performances and it is less sensitive to the choice of the “calibration” parameters (Z_score_ for the SVM based and prominence for the peak finder).

It has to be highlighted that the present apparatus, based on elastic LiDAR, may be sensitive to many other variations in the atmosphere, which may lead to an increase of the backscattering variations (especially low-height clouds and fogs), involving an increase of false positives. More sophisticated approaches may be used. For example, one may use the polarization-based setup, a multiwavelength LiDAR or a DIAL setup [7,22].

## Figures and Tables

**Table 1:** Characteristics of LiDAR main components.

Light Source			
Laser type	ND:YaG	Pulse width	8 ns
Functioning	Pulsed	Beam diameter	5 mm
Mean wavelength	1064 nm	Beam divergence	4 mrad
Max repetition rate	10 Hz		
**Telescope**			
Nominal focal length	1300 mm	Primary mirror diameter	102 mm
f-number	f/12.7	Field of view	1.2 mrad
**Photodiode**			
Time response	5 ns		
Gain	100		
Quantum Efficiency	38%		
**DAQ—Card PXI 5122**			
Resolution	14 bit		
Sampling rate	100 Msample/s		
